# Egr-1 mediates the suppressive effect of IL-1 on PPARg expression in human OA chondrocytes

**DOI:** 10.1186/ar3613

**Published:** 2012-02-09

**Authors:** Sarah S Nebbaki, Fatima Ezzahra El Mansouri, Mohamed Benderdour, Johanne Martel-Pelletier, Jean-Pierre Pelletier, Hassan Fahmi

**Affiliations:** 1 Osteoarthritis Research Unit, Montreal, H2L 4M1, Canada

## Background

Peroxisome proliferator-activated receptor gamma (PPARg) is a ligand activated transcription factor and member the nuclear hormone receptor superfamily. Several lines of evidence indicate that PPARg have protective effects in osteoarthritis (OA). Indeed, PPARg has been shown to down-regulate several inflammatory and catabolic responses in articular joint cells and to be protective in animal models of OA. We have previously shown that IL-1 down-regulated PPARg expression in OA chondrocytes. In the present study we will investigate the mechanisms underlying this effect of IL-1.

## Materials and methods

Chondrocytes were stimulated with IL-1, and the level of PPARg and Egr-1 protein and mRNA were evaluated using Western blotting and real-time reverse-transcription polymerase chain reaction, respectively. The PPARg promoter activity was analyzed in transient transfection experiments. Egr-1 recruitment to the PPARg promoter was evaluated using chromatin immunoprecipitation (ChIP) assays.

## Results

We demonstrated that the suppressive effect of IL-1 on PPARg expression requires de novo protein synthesis and was concomitant with the induction of the transcription factor Egr-1. ChIP analyses revealed that IL-1 induced Egr-1 recruitment at the PPARg promoter. IL-1 inhibited the activity of PPARg promoter and overexpression of Egr-1 potentiated the inhibitory effect of IL-1, suggesting that Egr-1 may mediate the suppressive effect of IL-1.

## Conclusions

These results indicate that Egr-1 contributes to IL-1-mediated down-regulation of PPARg expression in OA chondrocytes and suggest that this pathway could be a potential target for pharmacologic intervention in the treatment of OA and possibly other arthritic diseases.

